# Endocytic Adaptor Protein Epsin Is Elevated in Prostate Cancer and Required for Cancer Progression

**DOI:** 10.1155/2013/420597

**Published:** 2013-04-04

**Authors:** Kandice L. Tessneer, Satish Pasula, Xiaofeng Cai, Yunzhou Dong, Xiaolei Liu, Lili Yu, Scott Hahn, John McManus, Yiyuan Chen, Baojun Chang, Hong Chen

**Affiliations:** ^1^Cardiovascular Biology Program, Oklahoma Medical Research Foundation, Oklahoma City, OK 73104, USA; ^2^Biochemistry and Molecular Biology Department, University of Oklahoma Health Science Center, Oklahoma City, OK 73104, USA

## Abstract

Epsins have an important role in mediating clathrin-mediated endocytosis of ubiquitinated cell surface receptors. The potential role for epsins in tumorigenesis and cancer metastasis by regulating intracellular signaling pathways has largely not been explored. Epsins are reportedly upregulated in several types of cancer including human skin, lung, and canine mammary cancers. However, whether their expression is elevated in prostate cancer is unknown. In this study, we investigated the potential role of epsins in prostate tumorigenesis using the wild type or epsin-deficient human prostate cancer cells, LNCaP, in a human xenograft model, and the spontaneous TRAMP mouse model in wild type or epsin-deficient background. Here, we reported that the expression of epsins 1 and 2 is upregulated in both human and mouse prostate cancer cells and cancerous tissues. Consistent with upregulation of epsins in prostate tumors, we discovered that depletion of epsins impaired tumor growth in both the human LNCaP xenograft and the TRAMP mouse prostate. Furthermore, epsin depletion significantly prolonged survival in the TRAMP mouse model. In summary, our findings suggest that epsins may act as oncogenic proteins to promote prostate tumorigenesis and that depletion or inhibition of epsins may provide a novel therapeutic target for future prostate cancer therapies.

## 1. Introduction

Solid tumors, such as those in prostate cancer, contribute the majority of all cancers and result in significant distant tumor metastasis to vital organs such as the lungs, brain, and bones [1, 2]. Prostate cancer contributes significantly to the morbidity and mortality of men in the United States [3]. Advanced prostate cancer is associated with significant mortality because the cancer metastasizes and spreads throughout the body, making recovery nearly impossible [1, 4]. The high rates of prostate cancer metastasis are, in part, caused by aggressive primary tumor growth in prostate [5]. Prostate tumorigenesis is a result of several upregulated signaling pathways, including Notch, EGF, FGF, and Wnt signaling, which promote tumor cell proliferation [6–11]. Understanding the mechanisms responsible for upregulated signaling during early tumorigenesis is an important step in identifying key regulators and potential therapeutic targets. More importantly, targeting early stages of tumorigenesis will facilitate stabilization of rapid growing tumors, leading to effective surgical removal of primary tumors and inhibition of further tumor metastasis.

Epsins are endocytic adaptor proteins that regulate clathrin-mediated endocytosis of cell surface receptors by binding ubiquitin moieties on the receptor cytoplasmic tail [12–20]. Mammals express three epsins; epsins 1 and 2 are ubiquitously expressed in all tissues while epsin 3 is exclusively expressed in the stomach [14, 20, 21]. Epsins 1 and 2 likely play redundant functions in mediating endocytosis because a single deletion of either epsin 1 or 2 does not present with a significant phenotype [22, 23]. However, epsins 1 and 2 are essential during development because deletion of both alleles (DKO) resulted in embryonic lethality (E9.5) [22]. Embryonic lethality was due in large part to a defective vascular phenotype. We recently reported, using inducible epsin depletion, that postnatal loss of epsins 1 and 2 (iDKO) presented a normal phenotype, suggesting epsins play an important role in the development, but not the maintenance, of normal healthy tissues [23]. In contrast, endothelial cell-specific epsin depletion significantly impaired the growth of several tumor types as a result of aberrant tumor angiogenesis [23]. Solid tumorigenesis, including tumorigenesis of the prostate, is dependent on both tumor angiogenesis and tumor cell proliferation [5, 24]. Several signaling pathways involved in tumor cell proliferation involve clathrin-mediated receptor internalization and, therefore, may be mediated by epsins [11].

Elevated levels of epsins have been reported in human skin and lung cancers, as well as canine mammary cancers [23, 25–28]. Epsin overexpression promotes cancer cell invasion through the inhibition of Cdc42 and Rac1 GAP activity and by binding to RalBP1 [25, 27, 29–31]. Intriguingly, in this study, we have also discovered that expression of epsins is upregulated in both human and mouse prostate cancer cells and prostate cancer tissues. We have utilized both the human prostate LNCaP xenograft model [32] and the spontaneous TRAMP mouse model [33] to determine if epsin depletion affects prostate tumorigenesis. Our findings implicate epsins as oncogenic proteins whose expression is upregulated in prostate epithelium and facilitates growth and progression of prostate cancer. Furthermore, our data suggest that depletion or inhibition of epsins may provide novel therapeutic targets for solid tumor therapies.

## 2. Materials and Methods

### 2.1. Generation of Conditional Epn1^fl/fl^ Mice and Tamoxifen-Inducible Epsin DKO

We recently reported a strategy for generating a global epsins 1 and 2 double knockout (DKO) mouse model [22]. C57BL/6 background mice with epsin 1 flanked by loxP sites (Epn1^fl/fl^) on a global epsin-2-deficient background (Epn1^fl/fl^, Epn2^−/−^) were used to create iDKO mice by crossing Epn1^fl/fl^, Epn2^−/−^ mice with ER^T2^Cre deleter mice that express Cre Recombinase in all cell types upon tamoxifen injection (iDKO) [23]. 

### 2.2. Prostate Tumor Model

We crossed iDKO and WT mice with Transgenic Adenocarcinoma of Mouse Prostate (TRAMP) mice on C57BL/6J background [33] to generate TRAMP-WT or TRAMP-iDKO mice. Ten-week-old, sex and genetic background matched TRAMP-iDKO or TRAMP-WT mice were IP-injected with 4-hydroxytamoxifen (150 *μ*g per 30 g of body weight) for five to seven consecutive days to induce deletion of epsin 1. Mortality of TRAMP-WT and TRAMP-iDKO mice was recorded and survival rates plotted as a Kaplan-Meier plot. Mice were also dissected at weeks 20, 24, 28, 32, and 36 and checked for prostate tumors. Tumor growth in two groups of mice was monitored by measuring tumor size with digital calipers. We recognized tumors more than 2 mm in diameter as positive and calculated tumor volume based on the formula: 0.5326 (length [mm] × width [mm]^2^). 

### 2.3. Antibodies and Reagents

 Polyclonal rabbit antibodies for epsins 1 and 2 were obtained as previously described [14, 17]; goat anti-epsin 1 and mouse anti-GAPDH were obtained from Santa Cruz. 4-hydroxytamoxifen was from Sigma.

### 2.4. Cell Culture

Human LNCaP cells (ATCC) were cultured at 37°C with 5% CO_2_ in RPMI 1640 medium supplemented with 10% fetal bovine serum (FBS) and 1% penicillin/streptomycin.

### 2.5. Western Blot Analyses

Human LNCaP cells were treated with either control GFP lentivirus or epsins 1 and 2 shRNA lentivirus for 2 days then incubated with fresh media for an additional 2 days. Cells were lysed with RIPA Buffer (1%Triton X-100/0.1%SDS/0.5% sodium deoxycholic acid/5 mM tetrasodium pyrophosphate/50 mM sodium fluoride/5 mM EDTA/150 mM NaCl/25 mM Tris, pH 7.5/5 mM Na_3_VO_4_/5 mM N-ethylmaleimide, and protease inhibitor cocktail) then the deletion of epsins 1 and 2 was analyzed by western blot using goat anti-epsin 1 and rabbit anti-epsin 2. Deletion of epsins 1 and 2 in normal prostate tissues and prostate tissue with tumor was analyzed by western blotting homogenized tissues using rabbit anti-epsin 1 and rabbit anti-epsin 2 antibodies. 

### 2.6. Tumor Implantation

 To assess tumor growth, we subcutaneously implanted human prostate WT or epsins- 1- and 2-deficient LNCaP cells (3 × 10^6^ cells/tumor) in twelve-week-old SCID mice. We estimated the time of tumor appearance and monitored the tumor growth in two groups of mice by measuring tumor size with digital calipers. We recognized tumors more than 2 mm in diameter as positive and calculated tumor volume based on the formula: 0.5326 (length [mm] × width [mm]^2^). SCID mice were obtained from The Jackson Laboratory, Maine, USA.

### 2.7. Immunohistochemistry and Immunofluorescence of Tissue Samples

Immunohistochemistry and immunofluorescence were performed as described with modifications [13, 22]. Human prostate frozen tissue samples with stage 2C tumors, and their corresponding surrounding nonneoplastic (normal) tissues, used for epsins 1 and 2 immunofluorescence studies were purchased as AccuMax array from ISU ABXIS. Images were collected and quantified using an Olympus Spinning Disc Confocal Microscope equipped with 20x objective and Slidebook 5.0 software.

### 2.8. Study Approval

All animal studies were performed in compliance with institutional guidelines and were approved by Institutional Animal Care and Use Committee (IACUC), Oklahoma Medical Research Foundation, Oklahoma City.

### 2.9. Statistical Analysis

 Data were shown as mean ± SEM. Data was analyzed by the two-tailed student's *t*-test or ANOVA, where appropriate. The Wilcoxon signed-rank test was used to compare data that did not satisfy the student's *t* test or ANOVA. *P* value ≤0.05 was considered significant.

## 3. Results

### 3.1. Upregulated Epsins 1 and 2 in Human Prostate Carcinoma

**Figure 1:**
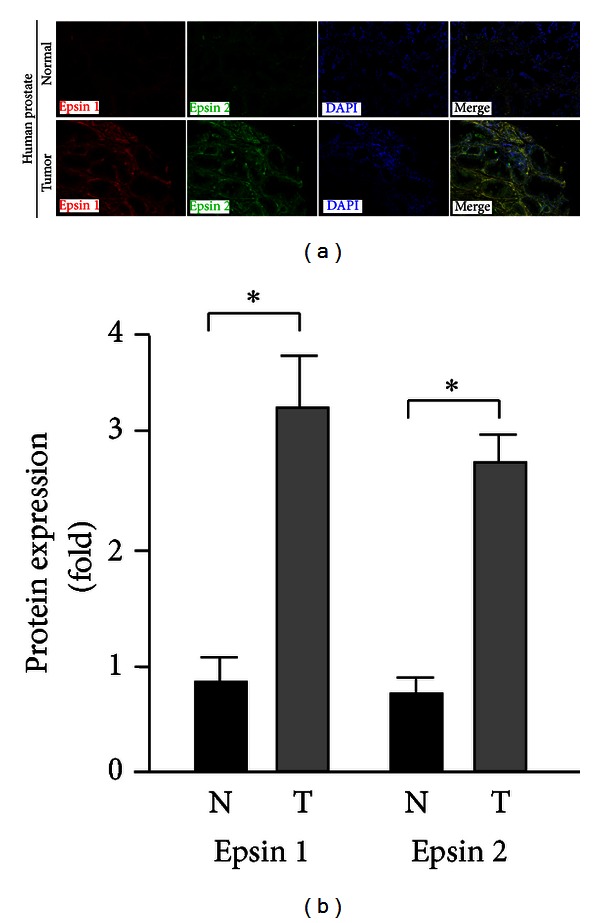
Expression of epsins 1 and 2 is increased in human prostate cancer tissues. Protein levels of epsins 1 and 2 were analyzed by immunostaining paired nonneoplastic (normal) and tumorigenic human prostate tissues. (a) Representative image from a single paired sample. (b) Quantification of fluorescence intensity from *n* = 6. *indicates *P* value <0.05.

Epsins are reportedly upregulated in several types of cancer [23, 25, 26]. To begin investigating a potential correlation between epsins and the development of prostate cancer, we measured the protein levels of epsins 1 and 2 in nonneoplastic (normal) human prostate and tumorigenic human prostate tissues via immunofluorescent staining. We found that epsins 1 and 2 were present at low levels in epithelial cells of acini of normal human prostate tissue (Figure 1(a)). Furthermore, epithelial epsins 1 and 2 were significantly upregulated in the human prostate tumor tissues (Figures 1(a) and 1(b)). These data demonstrate a positive correlation between protein levels of epsins and prostate tumorigenesis. 

### 3.2. Upregulated Epsins 1 and 2 Expression in Prostate Carcinoma of Spontaneous TRAMP Model

**Figure 2:**
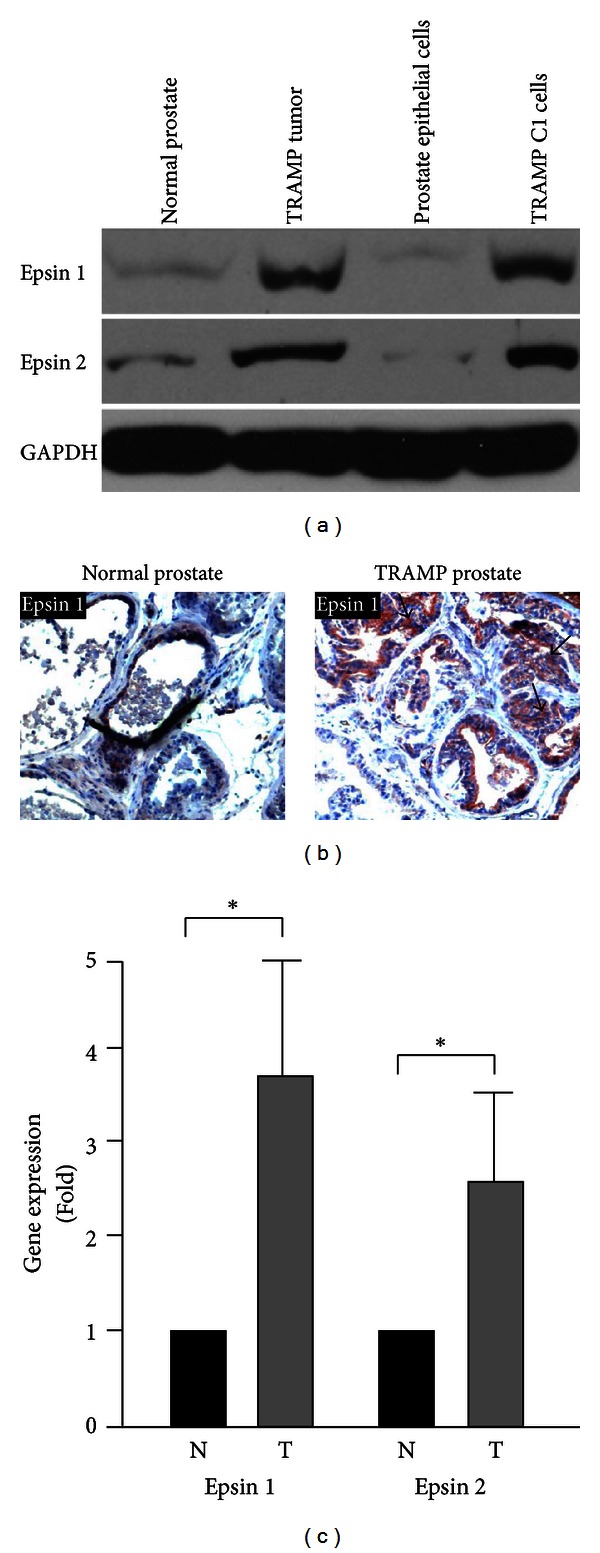
Expression of epsins 1 and 2 is increased in prostate tumor compared to normal prostate tissues in mice. (a) Protein levels of epsins 1 and 2 in the indicated normal mouse prostate or TRAMP prostate tumor tissues, as well as normal prostate epithelial cells and TRAMP C1 cells, were analyzed by western blotting. (b) IHC staining for epsin 1 in normal prostate and prostate tumor from TRAMP mice. Arrows indicate prostate cancer cells with upregulated epsin 1 protein expression. (c) qRT-PCR revealed increased expression of epsins 1 and 2 in normal (N) and prostate tumor (T) samples from TRAMP mice. *n* ≥ 5 per group in all panels. *indicates *P* value <0.05.

To investigate whether the increased proteins detected in human prostate cancer tissues were a result of increased epsins 1 and 2 gene expression, we employed the spontaneous Transgenic Adenocarcinoma of Mouse Prostate (TRAMP) mouse model [33–36]. We first confirmed, via western blotting and immunohistochemistry (IHC), that epsins 1 and/or 2 protein levels were upregulated in prostate tumors extracted from TRAMP mice (Figures 2(a)-2(b)). Then, we used quantitative real-time PCR (qRT-PCR) to investigate the gene expression of epsins 1 and 2. Consistent with increased protein detection, we found that the gene expression of epsins 1 and 2 increased by 3.5- or 2.5-fold, respectively, in extracted TRAMP prostate tumors, compared to normal prostate (Figure 2(c)). These data demonstrate that the expression of epsins 1 and 2 is upregulated in prostate tumors *in vivo* and further support a correlation between increased epsins 1 and 2 and prostate tumorigenesis. 

### 3.3. Epsins 1 and 2 Depletion Impairs Prostate Cancer Progression in Xenograft Model

**Figure 3:**
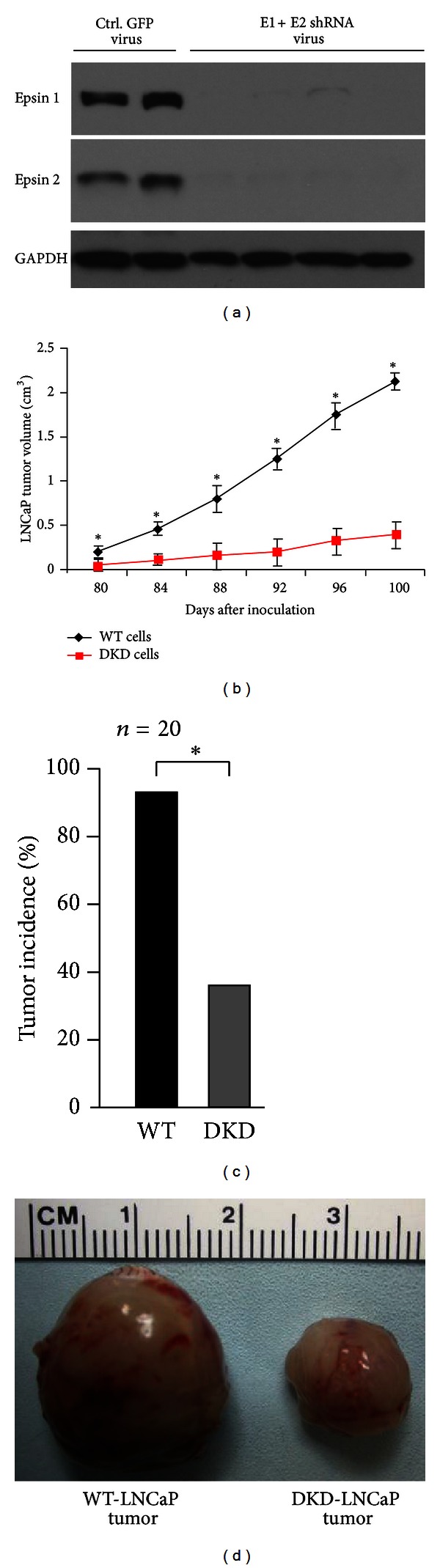
Epsins-1- and 2-deficient human prostate cells result in retarded tumor growth. (a) Lysates of LNCaP cells treated with either control GFP lentivirus or epsins-1- and 2-shRNA lentivirus were analyzed for epsins 1 and 2 depletion by western blotting. (b) WT or epsins-1- and 2-deficient LNCaP cells (DKD) were injected subcutaneously into SCID mice and LNCaP tumor size measured from days 80 to 100 afte-inoculation. (c) LNCaP tumor incidence. (d) Representative WT or epsins-1- and 2-deficient LNCaP tumors dissected out after 100 days of postinoculation. *n* = 5 in a, *n* = 20 in (b)–(d). *indicates *P*-value <0.05.

 Human prostate cancer LNCaP cells are a common model for prostate cancer progression because they share many characteristics of early prostate cancer progression including low tumorigenesis and androgen sensitivity [7, 32, 37, 38]. We transfected LNCaP cells with control GFP lentivirus or epsins 1 and 2 shRNA lentivirus and confirmed epsin depletion by western bot (Figure 3(a)). We subcutaneously inoculated control or epsin-depleted LNCaP cells into SCID mice to determine if epsin depletion would alter LNCaP tumorigenesis. LNCaP tumors were removed and measured from days 80 to 100 after-inoculation. We found that knockdown of epsins significantly impaired LNCaP tumor growth (Figures 3(b)–3(d)) and reduced tumor incidence (Figure 3(c)). These data suggest an oncogenic role for epsins in prostate tumorigenesis and cancer progression in a human xenograft model. 

### 3.4. Epsins 1 and 2 Depletion Impairs Prostate Cancer Progression in Spontaneous TRAMP Model

**Figure 4:**
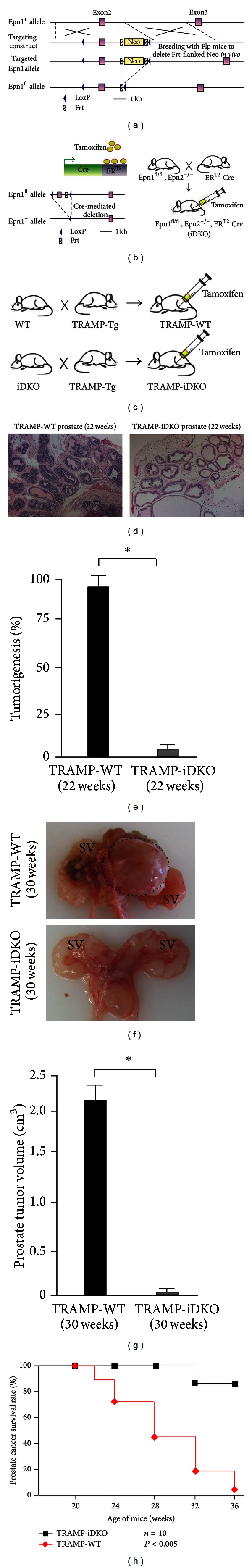
Loss of epsins 1 and 2 results in reduced tumor growth and increased survival rate in spontaneous TRAMP tumor model. (a) Generation of Epn1^fl/fl^. The diagram shows homologous recombination of the floxed gene-targeting vector at the Epsin 1 (Epn1) locus. Wild type Epn1 allele, top row; targeting construct, second row; targeted Epn1 allele, third row; and Epn1 floxed allele without Neo cassette (Epn1^fl/fl^), fourth row. (b) Strategy to generate tamoxifen inducible DKO (iDKO) by crossing Epn1^fl/fl^; Epn2^−/−^ with ER^T2^Cre deleter mice. (c) Strategy to generate TRAMP-WT and TRAMP-iDKO mice by crossing TRAMP-tg mice with WT or Epn1^fl/fl^; and Epn2^−/−^; ER^T2^Cre (iDKO) mice. (d) H&E staining of prostate tissue from TRAMP-WT and TRAMP-iDKO mice dissected at 22 weeks. (e) Percentage of prostate tumorigenesis in TRAMP-WT and TRAMP-iDKO mice dissected at 22 weeks. (f) Male urogenital tract of TRAMP-WT and TRAMP-iDKO mice show tumor in prostate of TRAMP-WT but not in TRAMP-iDKO. Black dotted lines indicate tumors. Mice were sacrificed at 30 weeks. (g) Quantification of prostate tumor volume from TRAMP-WT and TRAMP-iDKO mice dissected at 30 weeks. (h) Percentage of male mice surviving in presence and absence of epsins 1 and 2 as a Kaplan-Meier plot. *n* = 20 in (d)–(g) and *n* = 10 in (h). *indicates *P*-value <0.05.

 To further investigate the role of epsins 1 and 2 in the development and progression of prostate cancer in a spontaneous cancer model, we generated an epsin-deficient TRAMP mouse model (Figure 4). First, we created mice with a conditional epsin 1 allele (Epn1^fl/fl^) (Figure 4(a)) then crossed them with epsin 2 null mice (Epn2^−/−^), thereby creating Epn1^fl/fl^, Epn2^−/−^ mice [22]. To obtain epsins 1 and 2 inducible-double knockout mice (iDKO), the Epn1^fl/fl^, Epn2^−/−^ mice were crossed with mice expressing tamoxifen-inducible Cre Recombinase (ER^T2^Cre) (Figure 4(b)). The Epn1^fl/fl^, Epn2^−/−^, ER^T2^Cre mice were further crossed with TRAMP mice to generate TRAMP, Epn1^fl/fl^, Epn2^−/−^, ER^T2^Cre mice. Ten-week old TRAMP, Epn1^fl/fl^, Epn2^−/−^, ER^T2^Cre mice, sex and genetic background matched to TRAMP-WT mice, were treated every day for two weeks with 5 mg of 4-hydroxytamoxifen per kg body weight via intraperitoneal injection (I.P.) to induce epsin 1 deletion (TRAMP-iDKO) (Figure 4(c)). To examine the effects of epsin deletion on spontaneous prostate tumor progression in the TRAMP background, we examined tumor development in the male urogenital tract of TRAMP-WT and TRAMP-iDKO mice. We found that similar to the LNCaP model, depletion of epsins impaired spontaneous prostate tumorigenesis (Figures 4(d)–4(g)). Furthermore, epsin deletion in TRAMP mice significantly improved prostate cancer survival rates (Figure 4(h)). In summary, our data demonstrates that epsins are upregulated in, and likely facilitate tumorigenesis of, the prostate. Furthermore, interfering with epsins may protect against the growth and progression of prostate cancer. 

## 4. Discussion

Studies investigating the physiological and pathological roles of epsins have revealed their importance in many biological processes [16, 20, 22, 23, 26]. However, the full extent with which epsins are involved in the pathological development of diseases, such as cancer, or their tissue and cell type-specific contributions is still largely unknown. In this study, we investigated the potential role of epsins in the development and progression of prostate cancer. Our findings suggest that epsins act as oncogenic proteins to promote prostate tumor growth and progression. Furthermore, our results suggest that inhibition of epsins may provide a mechanism to impair the onset and progression of prostate cancer. 

We chose to study the effects of epsin depletion in two common models of prostate cancer, subcutaneous xenograft of the LNCaP human prostate cancer cells and the genetic TRAMP mouse model. In both models we found that depletion of epsins impaired the growth of prostate tumors. In addition, using the TRAMP mouse model, which closely mimics the spontaneous growth and progression of prostate tumors in human prostate cancer [35, 36, 39], we were able to show that depletion of epsins significantly prolonged prostate cancer survival. Increased survival is likely a result of lower tumor incidence, impaired tumor growth, and reduced metastasis.

Interestingly, both human prostate tumors and TRAMP mouse prostate tumors exhibited elevated epsin proteins. Furthermore, using the TRAMP model, we determined that increased epsin proteins were due to significant increases in the gene expression of epsins 1 and 2 in tumor tissue. It is not clear from these results whether upregulated epsin expression is a cause or consequence of tumorigenesis. However, the fact that loss of epsins protected against tumor growth suggests the former, rather than the latter, is likely. The transcriptional regulation of epsins is not currently understood but this data suggests it may provide important information about what mediates early prostate cancer progression. 

Lastly, epsins are characterized as endocytic adaptors that regulate the internalization of several ubiquitinated receptors [20]. Although not addressed herein, it is tempting to speculate that the overexpression of epsins may play an important role in upregulating signaling pathways dependent on receptor internalization. Alternatively, the multidomain nature of epsins may facilitate the stabilization of cell surface receptor complexes, thereby providing a mechanism to prolong signals that promote tumor growth. In summary, epsins are upregulated in human and mouse prostate carcinoma and this upregulation positively correlates with tumor growth. Loss of epsins impaired prostate tumor progression and promoted survival in the TRAMP mouse model. Our results suggest targeted inhibition of epsins may provide novel therapeutics to combat prostate cancer.
